# *In situ* detection of activation of CAPN3, a responsible gene product for LGMDR1, in mouse skeletal myotubes

**DOI:** 10.1016/j.jbc.2025.108536

**Published:** 2025-04-23

**Authors:** Chihiro Hisatsune, Fumiko Shinkai-Ouchi, Shoji Hata, Yasuko Ono

**Affiliations:** Calpain Project, Tokyo Metropolitan Institute of Medical Science, Tokyo, Japan

**Keywords:** calpain, muscular dystrophy, calcium, skeletal muscle, protein translocation

## Abstract

CAPN3/calpain-3/p94, a muscle-specific Ca^2+^-dependent cysteine protease, is responsible for limb-girdle muscular dystrophy R1 (LGMDR1), an autosomal recessive muscular dystrophy. However, the activation mechanism and physiological function of CAPN3 in skeletal muscles remain unknown. Here, we capture the *in situ* activation of CAPN3 in cultured mouse skeletal myotubes. Using our newly developed antibody, which specifically recognizes CAPN3 autolytic processing, we succeeded in differentiating WT CAPN3 from a protease-inactive CAPN3 mutant by immunostaining. We further demonstrated that CAPN3 predominantly localized at the M-bands of cultured skeletal myotubes at rest and translocated to the cytoplasm after activation by stimulation with ouabain, a cardiotonic steroid. This event requires a small but long-lasting cytoplasmic increase in Ca^2+^ levels, which is sufficient for the activation of CAPN3 but not of calpain-1/CAPN1. Activated CAPN3 digests the cytoskeletal proteins spectrin and talin. Thus, we successfully visualized the intracellular dynamics of endogenous CAPN3 in cultured skeletal muscles after activation by ouabain and demonstrated the subsequent processing of endogenous substrates in living cells. Our study will help understand the physiological functions of CAPN3 in skeletal muscles and the pathophysiological mechanisms of limb-girdle muscular dystrophy R1.

Limb-girdle muscular dystrophy type R1 (LGMDR1) is an autosomal recessive disease characterized by progressive atrophy of the proximal limb muscles, including the shoulder girdle muscles (1). The causal gene is *CAPN3*, which encodes a Ca^2+^-dependent cysteine protease (94 kDa) that is predominantly expressed in skeletal muscles (2). CAPN3 belongs to the classical calpain family, which is composed of three conserved domains: a calpain-type cysteine protease conserved (CysPc) domain containing protease core subdomains 1 and 2 (PC1 and PC2), a calpain-type β-sandwich (CBSW) domain, and a penta EF hand (PEF) domain (3, 4, 5). In addition to these conventional domains, it contains three specific insertion sequences: NS at the NH_2_ terminus, IS1 within the PC2 domain, and IS2 between the CBSW and PEF domains (2). These insertion sequences render CAPN3 inactive under resting conditions, and the autolytic removal of IS1 is necessary for CAPN3 to form a catalytic active center between the produced NH_2_ terminal (∼30 kDa) and the COOH-terminal fragment (58 kDa), enabling it to hydrolyze other substrates (6, 7, 8, 9, 10, 11).

To date, the autolysis of CAPN3, namely CAPN3 activation, has been determined using western blotting, which allows for the differentiation of the active-form CAPN3 from its inactive-form based on their reduced molecular size. However, western blotting cannot show the spatial information, and the location where CAPN3 undergoes autolysis cannot be determined. Given that CAPN3 activation is strongly correlated to the autolytic processing within the IS1 region (11), any device that detects the IS1 autolysis *in situ* would advance our understanding of the CAPN3 activation mechanism and physiological functions in skeletal muscles as well as elucidate the pathogenesis of LGMDR1.

In this study, we developed a novel antibody that specifically detects the autolysis of CAPN3 to visualize active form of CAPN3 in living cells. The developed antibody recognizes the COOH terminus of an autolytic cleavage fragment within the IS1 region of CAPN3, and therefore detects only the WT CAPN3, but not the protease-inactive CAPN3 mutant (CAPN3:C129S (CS)) on immunostaining. This antibody clearly illustrates the activation process of CAPN3 in cultured skeletal muscles upon ouabain treatment: activation at the M-bands and subsequent transition into the cytoplasm within the sarcomeres. We also demonstrated that ouabain caused a small, but sustained increase in cytosolic Ca^2+^ levels, sufficient for CAPN3 activation but not for CAPN1, a ubiquitous calpain. Thus, we examined the subcellular dynamics of CAPN3 following activation and identified two cytoskeletal proteins, spectrin and talin, as genuine CAPN3 substrates in living mouse skeletal myotubes.

## Results

### Production of antibody that detects autolytic cleavage of CAPN3

**Figure 1:**
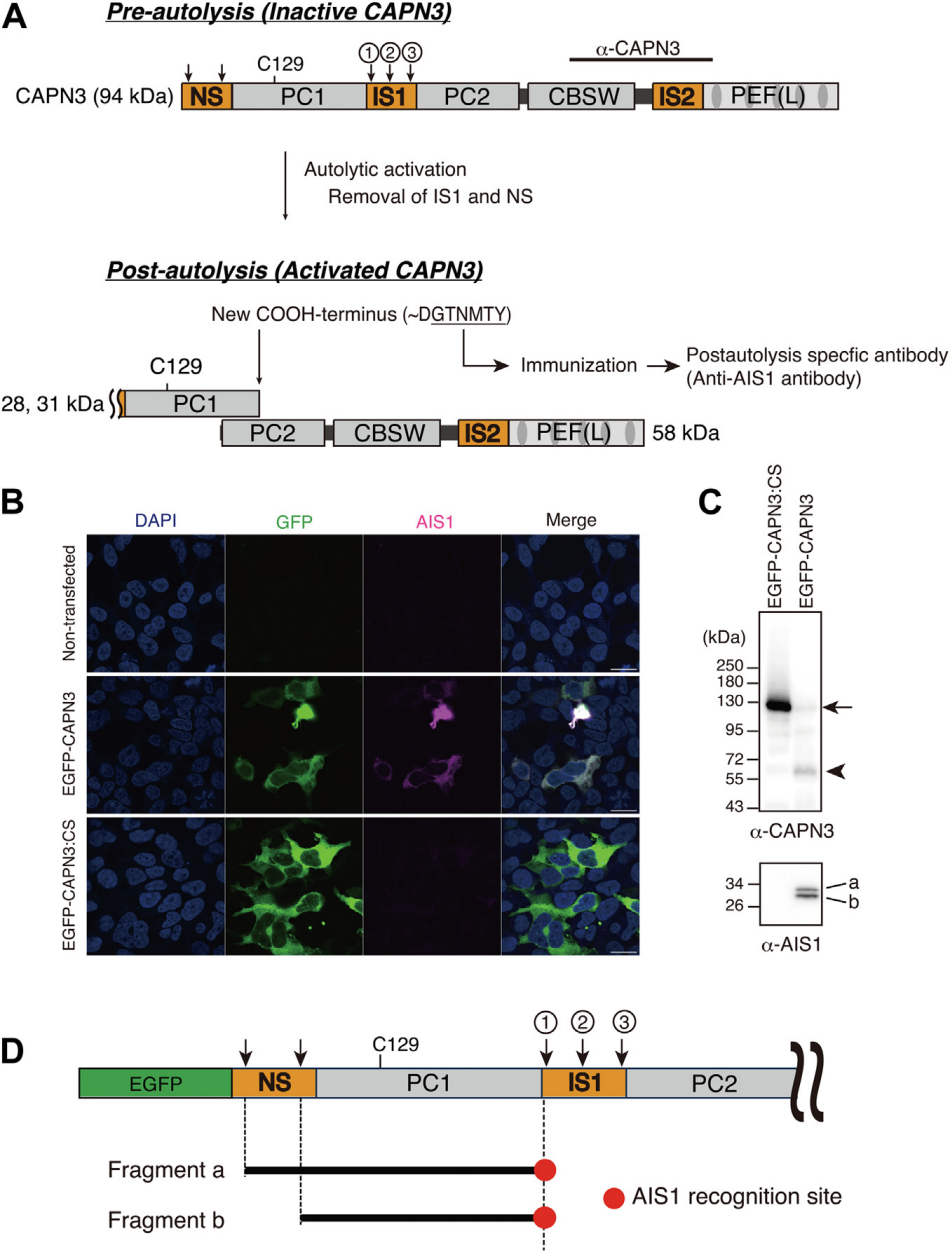
**Production of an antibody recognizing an autoprocessing site within the IS1 region of human CAPN3**. *A*, schematic illustration showing autoprocessing of CAPN3 and a synthesized peptide used for rabbit immunization. CBSW, calpain-type β-sandwich. IS, internal sequence. NS, N-terminal sequence. PC, protease core. PEF, penta EF motif. C129, H334, and N358 are catalytic centers. One, two, and three indicate the cleavage sites within the IS1 region. The *bar* indicates the antigen region (488–666 amino acids encoded by BC146672) of a commercial polyclonal anti-CAPN3 antibody (Proteintech, 284SS76-1-AP). *B*, immunostaining of HEK293T cells transiently expressing EGFP-CAPN3 or CAPN3:C129S (CS) with anti-AIS1 (*magenta*). EGFP (*green*). DAPI (*blue*). *C*, western blot of the cell lysates of HEK293T cells transiently expressing EGFP-CAPN3 and CAPN3:CS with anti-CAPN3 (*upper* panel) and anti-AIS1 (*lower* panel) antibody. An *arrow* shows the full-length of EGFP-CAPN3CS (130 kDa), and an *arrowhead* indicates the 58 kDa autocleaved fragment of EGFP-CAPN3. The 130 kDa band of the full-length of EGFP-CAPN3 is faint, because of rapid autolysis. *D*, schematic illustration of autolytic fragments and the recognition site of anti-AIS1 antibody. The two fragments (a and b) recognized by the anti-AIS1 antibody are approximately 30 kDa. AIS1, autolytic site within IS1; DAPI, 4′,6-diamidino-2-phenylindole; CAPN3, calpain 3.

CAPN3 has three unique sequences, NS, IS1, and IS2, compared to conventional CAPN1 and 2 (Fig. 1*A*). In particular, the presence of the IS1 amino acid sequence in the PC2 makes CAPN3 inactive in the naïve form, and requires the removal of IS1 by autolysis to become proteolytically active (6, 7, 8, 9, 10, 11). Therefore, we hypothesized that autolytic cleavage within the IS1 domain could be a good indicator of CAPN3 activation.

Three autocleavage sites exist within the IS1 sequence (Fig. 1*A*) (7, 12). We generated antibodies that specifically recognized the first cleavage site within the IS1 region to monitor autolysis of CAPN3 in this region. The rabbits were immunized with a synthetic peptide, “CGTNMTY” to generate an antibody that recognizes the COOH terminus of the peptide. Cleavage-site independent antibodies were removed from the immunized rabbit’s antisera using an affinity column with the synthetic peptide “CGTNMTYGTS.” The cleavage-site dependent antibody was then sequentially purified using an affinity column conjugated to the CGTNMTY peptide and named it as the “autolytic site within IS1 (AIS1) antibody.”

To confirm the specificity of our AIS1 antibody, HEK293T cells transiently expressing EGFP-tagged CAPN3 or the EGFP-tagged protease-inactive form of CAPN3 (EGFP-CAPN3:CS) were immunostained with the AIS1 antibody. As shown in Figure 1*B*, the AIS1 antibody showed clear immunosignals only in EGFP-CAPN3–transfected cells and not in EGFP-CAPN3:CS–transfected cells. Even on the high expression of EGFP-CAPN3:CS in HEK293T cells, the AIS1 antibody did not show any signal, indicating the specificity of the AIS1 antibody. On western blotting analysis of the transfected cell lysates using an anti-CAPN3 antibody (its recognition site is depicted in the Fig. 1*A*), we detected EGFP-CAPN3:CS at 130 kDa (arrow), whereas EGFP-CAPN3 was autolyzed and the cleaved 58 kDa fragment (arrowhead) was dominantly detected (Fig. 1*C*, upper panel). The AIS1 antibody detected two bands at approximately 30 kDa specifically in the EGFP-CAPN3, but not the EGFP-CAPN3:CS transfected cell lysates (Fig. 1*C*, lower panel). Based on their molecular sizes, these two bands were presumed to be autolytic fragments a and b (Fig. 1*D*).

### Detection of autolysis of LGMDR1 mutants by the anti-AIS1 antibody in living cells

**Figure 2:**
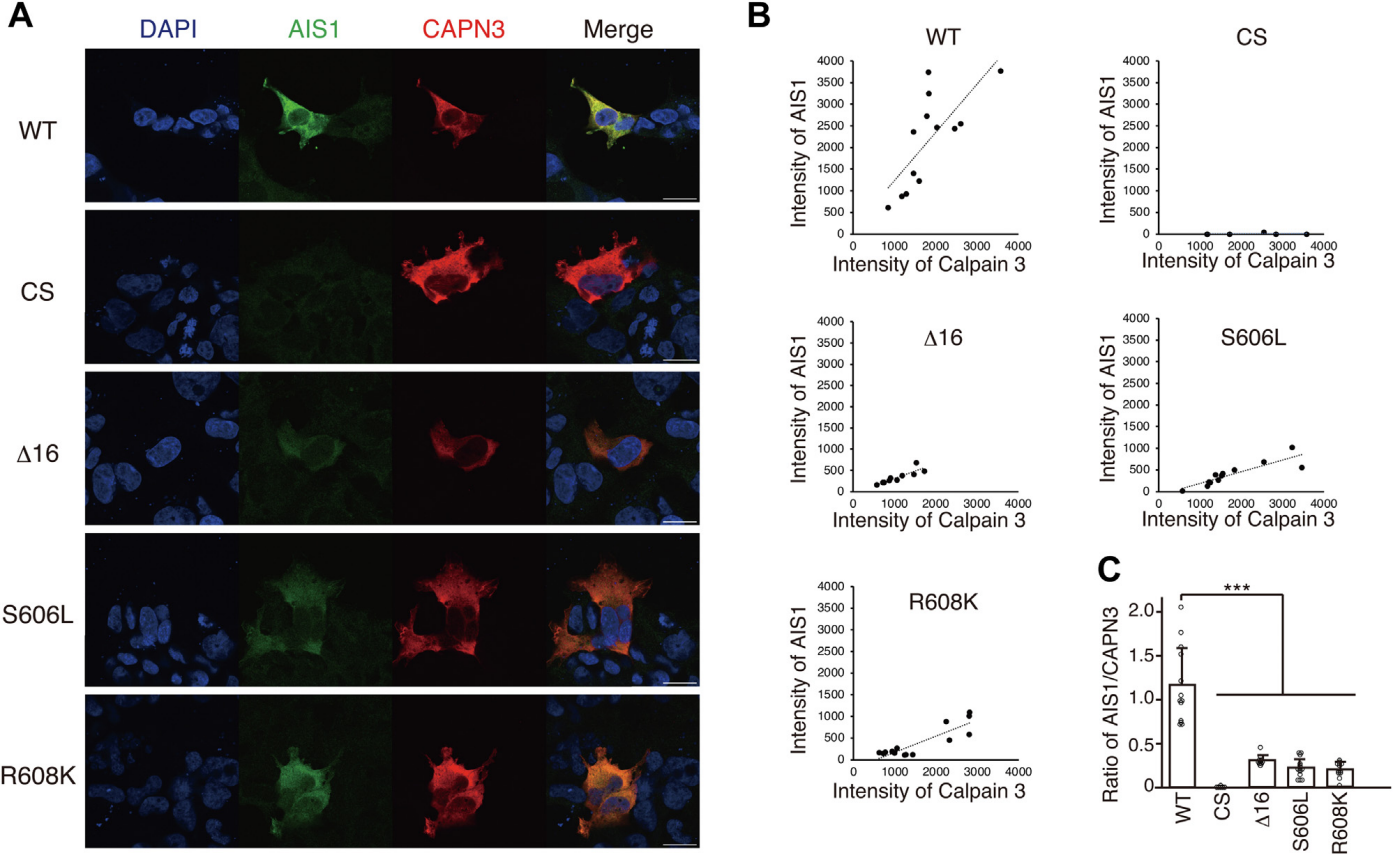
**Analysis of autolytic activity of LGMDR1 mutants by anti-AIS1 antibody**. *A*, immunostaining of COS-7 cells expressing WT CAPN3 and its mutants with CF488-conjugated anti-AIS1 (*green*) and CF555-conjugated anti-CAPN3 (*red*) antibody. The scale bar represents 20 μm. *B*, scatter plot of CAPN3 *versus* AIS1 intensities of WT CAPN3 and its mutants. *C*, mean ratio of AIS1 to CAPN3 intensity of WT and its mutants. N = 5 ∼ 14 cells. Each plot represents an individual cell value. Mean ± SD. ∗∗∗*p* < 0.001, one-way ANOVA with Dunnett’s multiple comparison test. AIS1, autolytic site within IS1; CAPN3, calpain 3.

Both our group and others previously reported that CAPN3 pathogenic mutants within the IS2 region, such as S606L, exhibit decreased autolytic activity (13, 14, 15). To examine whether the AIS1 antibody could discriminate the decreased autolytic activity of these mutants *in situ*, we stained COS-7 cells transiently expressing WT, CS, or several LGMDR1 pathogenic mutants of CAPN3 with anti-AIS1 and anti-CAPN3 antibodies covalently conjugated to CF488A dye and CF555 dye, respectively (Fig. S1). As shown in Figure 2*A*, AIS1 exhibited strong immunoreactivity in WT CAPN3–transfected cells, but was substantially weaker in S606L-, R608K-, and Δ16– transfected cells. Scatter plots of the immunostaining intensities of individual cells confirmed the decreased slope of the ratio (AIS1 versus CAPN3) in these mutants compared to the WT (Fig. 2, *B* and *C*). The decreased autolytic activities of the LGMDR1 mutants CAPN3:R608K and CAPN3:S606L were further verified by western blotting (13, 15) (Fig. S2). Thus, immunostaining of CAPN3 mutants with an anti-AIS1 antibody in combination with an anti-CAPN3 antibody revealed their decreased autolytic activity *in situ*.

### Ouabain treatment increases AIS1 immunosignals of CAPN3:S606L in HeLa cells in a Ca^2+^-dependent manner

**Figure 3:**
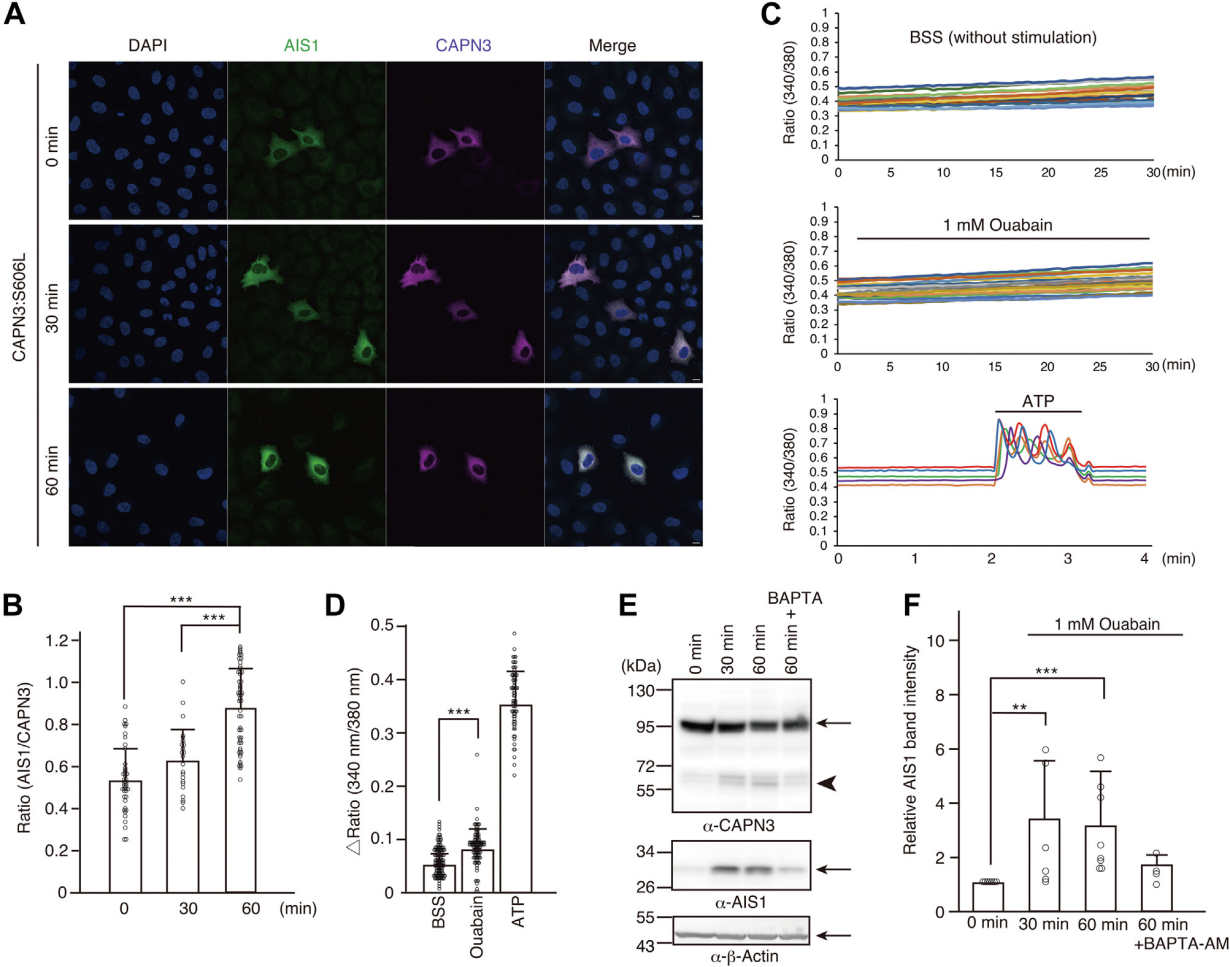
**Ouabain Ca^2+^ dependently activates CAPN3:S606L in HeLa cells**. *A*, immunostaining of HeLa cells transiently expressing CAPN3:S606L with anti-AIS1 (*green*) and anti-CAPN3 (*magenta*) antibody upon 1 mM ouabain stimulation. The scale bar represents 10 μm. *B*, change in cytosolic AIS1/CAPN3 immuno-intensity ratio of CAPN3:S606L-expressing cells after ouabain stimulation. Mean ± SD. Number of cells examined are 43, 23, and 52 for 0, 30, and 60 min, respectively. ∗∗∗*p* < 0.001, one-way ANOVA with Bonferroni’s test for multiple comparison. *C*, Ca^2+^ imaging of HeLa cells without BSS or with ouabain (1 mM) stimulation with a Ca^2+^ indicator, Fura 2. Ratio change of Fura 2 (340 nm/380 nm) is indicated. As a reference, Ca^2+^ signals of HeLa cells upon 10 μM ATP stimulation are also indicated. *D*, ratio change of Fura 2 (340 nm/380 nm) between 0 and 30 min with or without 1 mM ouabain stimulation. Mean ± SD. ∗∗∗*p* = 2.45 x 10^-9^, Two tailed student’s unpaired *t* test. As a reference, peak amplitude of the ratio changes of Fura 2 upon 10 μM ATP stimulation is also included in the figure. N = 123, 103, and 72 cells for BSS, ouabain, and ATP, respectively. *E*, pretreatment with a Ca^2+^ chelator BAPTA-AM (25 μM) inhibits the ouabain-induced autolysis of CAPN3:S606L in HeLa cells. The same membrane was probed with antibodies for CAPN3, AIS1, and β−actin. In the CAPN3 panel, an *arrow* shows the full-length of CAPN3:S606L (94 kDa), and an *arrowhead* indicates the autocleaved 58 kDa fragment of CAPN3:S606L. *F*, increase in the AIS1 band intensity of CAPN3:S606L after ouabain stimulation and its inhibition by BAPTA-AM treatment. The experiments were performed 8, 6, 8, and 4 times for each bar, respectively. Mean ± SD. ∗∗*p* = 0.01445, ∗∗∗*p* = 0.0037, Steel-Dwass test. AIS1, autolytic site within IS1; CAPN3, calpain 3.

We have previously shown that treatment with ouabain, a cardiotonic steroid that inhibits the Na^+^/K^+^ pump, increases the autolysis of CAPN3 in primary cultured skeletal myotubes (16). To test the generality of this phenomenon in other cell types, we examined whether the AIS1 signals of CAPN3:S606L in HeLa cells increased upon ouabain treatment. CAPN3:S606L, which exhibits decreased autolytic activity compared to WT CAPN3 was used, because WT CAPN3 remained completely activated in transfected HeLa cells, and the AIS1 immunosignals were high even before ouabain stimulation (Fig. 2). We transiently expressed CAPN3:S606L in HeLa cells, stimulated the cells with 1 mM ouabain, and coimmunostained the cells with CF555-linked anti-CAPN3 and CF488-linked anti-AIS1 antibodies. Although the AIS1 immunosignals of CAPN3:S606L were weak at 0 min, ouabain treatment gradually increased the AIS1 immunosignals in a time-dependent manner. Additionally, the ratio of AIS1 to CAPN3 intensity in the cytoplasm increased in a time-dependent manner (Fig. 3*B*). Thus, ouabain treatment activated CAPN3:S606L in HeLa cells, and the anti-AIS1 antibody could effectively detect the activation of CAPN3:S606L by ouabain upon immunostaining.

To examine the involvement of intracellular Ca^2+^ signals in the ouabain-induced CAPN3’s autolysis, we measured the Ca^2+^ signals in HeLa cells following ouabain treatment. HeLa cells were loaded with Fura 2-AM and the fluorescence ratio change (340 nm/380 nm) of Fura 2 in the absence or presence of ouabain was measured. No significant change in Ca^2+^ signals was observed. Instead, we observed a small but long-lasting increase in the Fura 2 ratio (Fig. 3*C*). The increase in the Fura 2 ratio (340 nm/380 nm) during the 30 min ouabain treatment was minimal but significant compared to nontreated control cells (BSS). As a reference, we also stimulated HeLa cells with 10 μM ATP and observed a large increase in the Fura 2 ratio (Fig. 3, *C* and *D*).

To further confirm the Ca^2+^ dependence of CAPN3:S606L activation upon ouabain treatment, we treated the cells with BAPTA-AM, a Ca^2+^ chelator, prior to ouabain stimulation. Ouabain treatment gradually increased the number of autolytic bands appearing around 58 kDa (Fig. 3*E*). Moreover, the band migrating around 30 kDa, detected using the anti-AIS1 antibody, also increased after stimulation. However, pretreatment of cells with BAPTA-AM inhibited the ouabain-induced autolysis of CAPN3:S606L. Quantification of the AIS1 band intensity confirmed that BAPTA-AM pretreatment decreased ouabain-induced autolysis of CAPN3:S606L (Fig. 3*F*). Although we previously reported that Na ^+^ can activate CAPN3 autolysis *in vitro* (16), the results of this study indicated that autolysis of CAPN3 by ouabain requires cytosolic Ca^2+^ increment and that CAPN3 is not a sodium dependent protease in living cells, as pointed out in a recent study (17).

### Ouabain treatment induces endogenous CAPN3 autolysis and its subsequent translocation from M-bands to the cytosol of sarcomeres

**Figure 4:**
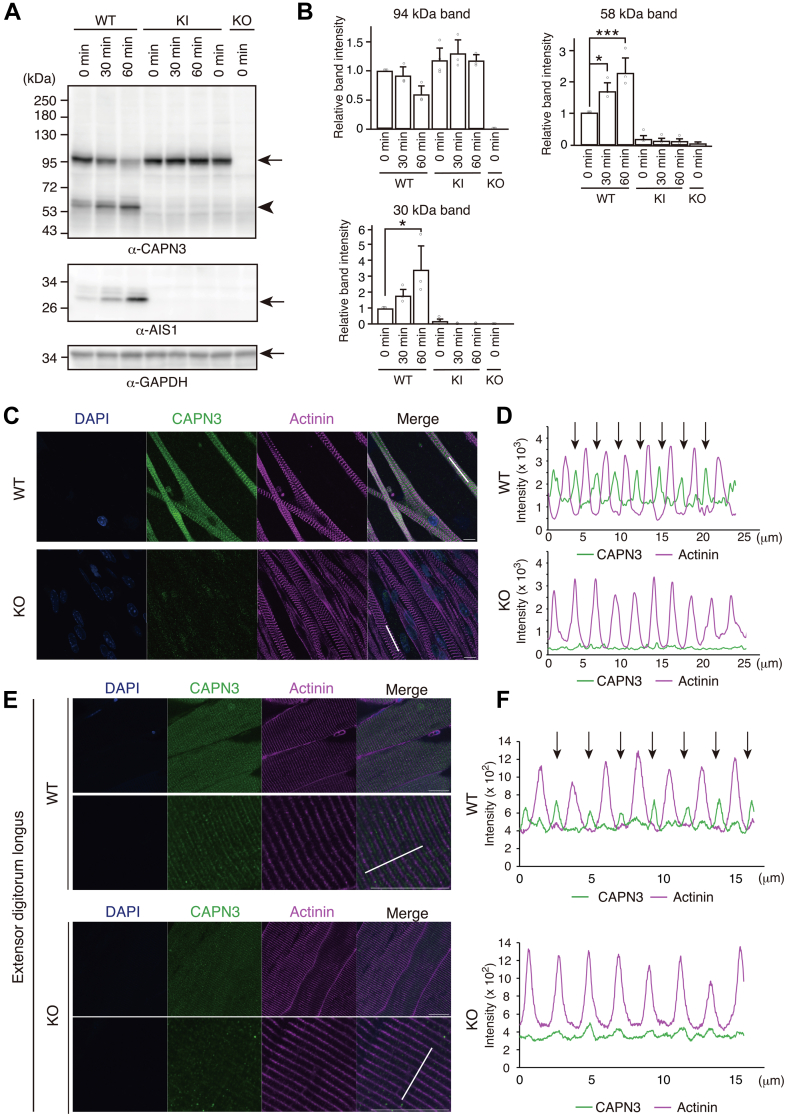
**Ouabain increases the autolysis of endogenous CAPN3 in mouse-cultured skeletal muscles**. *A*, autolysis of CAPN3 in cultured skeletal muscles (day 7 *in vitro* after differentiation) from WT, inactive-form knock-in (KI), and KO mice. The same membrane was probed with antibodies for CAPN3, AIS1, and GAPDH. In the upper CAPN3 panel, an *arrow* shows the full-length of CAPN3 (94 kDa) and an *arrowhead* indicates autocleaved 58 kDa fragment of CAPN3. *B*, changes in the relative band intensity of 94 kDa, 58 kDa, and 30 kDa in *A*. Mean ± SD. N = 3∼4. ∗*p* < 0.05, ∗∗∗*p* < 0.001, one-way ANOVA with Dunnett’s multiple comparison tes*t*. *C*, validation of the specificity of the anti-CAPN3 (Proteintech, 28476-1-AP) antibody on cultured CAPN3 KO skeletal myotubes. *Green*: CAPN3; *magenta*: Actinin (Z-band). The scale bar represents 10 μm. *D*, intensity profiles of CAPN3 and actinin signals on *white bars* (2 μm thick) in the panels of *C*. *Upper* panel: WT myotubes; *lower* panel: KO myotubes. *Arrows* indicate CAPN3 immunosignals at the M-bands. *E*, immunohistochemistry of EDLs from WT and CAPN3 KO mice with anti-CPAN3 (*green*) and anti-actinin (*magenta*, Z-band) antibody. The scale bar represents 20 μm. *F*, intensity profiles of CAPN3 and actinin signals on *white bars* (2 μm thick) in the left panel E. *Upper* panel: WT EDL; *lower* panel: KO EDL. *Arrows* indicate CAPN3 immunosignals at the M-bands. AIS1, autolytic site within IS1; CAPN3, calpain 3; EDL, extensor digitorum longus.

We examined the ouabain-induced autolysis of endogenous CAPN3 in cultured skeletal myotubes from WT, CAPN3:C129S knock-in (KI), and CAPN3 KO mice by western blotting. In skeletal myotubes from WT mice, ouabain decreased the amounts of the 94 kDa band of CAPN3, but increased the amount of autolytic 58 kDa bands in a time-dependent manner. The intensity of the AIS1 band also increased in a time-dependent manner (Fig. 4, *A* and *B*). In contrast, the 94 kDa band of cultured skeletal myotubes from KI mice remained unchanged, and the AIS1 bands did not appear in the lysates of KI myotubes. No band was detected in the cell lysates from cultured CAPN3 KO myotubes, confirming the specificity of the antibodies used in the experiments.

Several studies have examined the subcellular localization of CAPN3 in skeletal myotubes using immunostaining. However, no reports have demonstrated the specificity of these antibodies in CAPN3 KO samples. Therefore, we examined the quality of the CAPN3 antibody (Proteintech 28476-1-AP) used for immunostaining before examining the subcellular localization of CAPN3 in myotubes. CAPN3 signals at M-bands were the most prominent, in addition to faint diffuse CAPN3 signals in the cytoplasm of sarcomeres in resting cultured skeletal muscles from WT mice (Fig. 4*C*). These predominant CAPN3 signals were confirmed by line plots; CAPN3 signals were located at the center between actinin signals, namely, the Z-bands (Fig. 4*D*). Immunosignals in the M-bands and cytoplasm were diminished in cultured skeletal myotubes from CAPN3 KO mice, confirming the specificity of the CAPN3 antibody (Fig. 4, *C* and *D*, lower panels). We also examined the specificity of the anti-CAPN3 antibody in the skeletal muscle tissues of adult mice. CAPN3 signals at the M-bands were distinct in the extensor digitorum longus muscles of WT mice as observed through immunohistochemistry, but were abolished in CAPN3 KO mice (Fig. 4, *E* and *F*).

**Figure 5:**
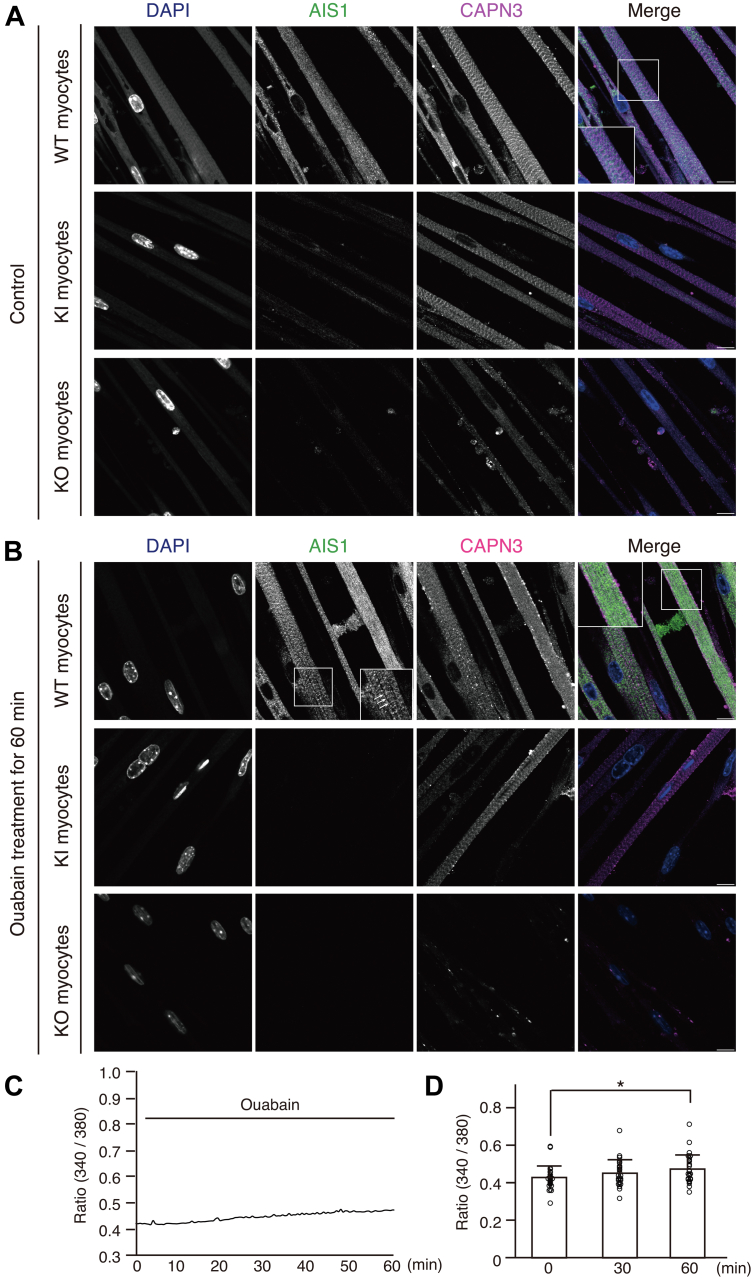
**Translocation of WT but not inactive-form of CAPN3 from the M-bands into the cytosol in cultured skeletal muscles after ouabain stimulation**. *A* and *B*, immunostaining of AIS1 and CAPN3 in cultured skeletal muscles from WT, KI, and KO mice before (*A*) and aftr 1 mM ouabain stimulation for 60 min (*B*). AIS1 (*green*), CAPN3 (*magenta*), and DAPI (*blue*). The *squares* show the magnified areas from the images. *Arrows* show faint striatal patterns of AIS1 signals in ouabain-treated WT myotubes. The scale bar represents 10 μm. *C*, ouabain-induced cytosolic Ca^2+^ levels in WT skeletal myotubes. Fura 2 ratio (340 nm/380 nm) for 60 min was plotted. *D*, time-dependent increase of Fura 2 ratio (340 nm/380 nm) following 1 mM ouabain stimulation. Mean ± SD. Number of cells analyzed were 28. ∗*p* = 0.0229, one-way ANOVA with Dunnett’s multiple comparison test. AIS1, autolytic site within IS1; CAPN3, calpain 3; DAPI, 4′,6-diamidino-2-phenylindole.

Next, we examined the subcellular localization of CAPN3 and the activated form of CAPN3 using CF555-linked anti-CAPN3 and CF488-linked anti-AIS1 antibodies (Fig. 5*A*) and after 60 min (Fig. 5*B*) of ouabain stimulation. In control cells, striated CAPN3 signals were predominantly observed at the M-bands. In addition, a small amount of dispersed cytosolic CAPN3 signal was detected. Weak AIS1 signals appeared diffused in the cytosol of resting WT skeletal myotubes. In KI myotubes, CAPN3 signals at the M-bands were similar to those in WT myotubes; however, AIS1 signals were rarely detected (Fig. 5*A*, middle panels). No signals for either AIS1 or CAPN3 were detected in the skeletal myotubes of KO mice (Fig. 5*A*, lower panels), confirming the specificity of the immunosignals.

Notably, when WT myotubes were stimulated with ouabain for 60 min, CAPN3 signals at the M-bands were scarcely visible and appeared to have translocated into the cytosol of the myotubes (Fig. 5, *A* and *B*, upper panels). In addition, the cytosolic diffused AIS1 signals became much stronger compared to those of the immunosignals in the resting cells (Fig. 5*B*, upper panels). In contrast, striated CAPN3 signals at the M-bands were still observed in KI myotubes even after ouabain stimulation (Fig. 5*B*, middle). No apparent change in the AIS1 signals in KI myotubes was observed before and after ouabain stimulation. Moreover, some AIS1 signals in ouabain-stimulated WT myotubes showed striated patterns, potentially at the M-bands (Fig. 5*B*, arrows). Taken together, these results suggest that CAPN3 is activated in M-bands and subsequently translocated into the cytoplasm of cultured skeletal myotubes upon ouabain stimulation. Furthermore, the data indicated that autolysis was essential for CAPN3’s translocation from the M-bands into the cytosol of sarcomeres in cultured skeletal muscles, as the inactive form of CAPN3KI was stably detected in a striated pattern.

We also examined Ca^2+^ signaling in skeletal myotubes following ouabain stimulation. A gradual increase in Ca^2+^ concentration in the cytosol upon ouabain stimulation was observed (Fig. 5, *C* and *D*). There was no significant change in the ouabain-induced Ca^2+^ signals in skeletal myotubes, similar to that observed in HeLa cells (Fig. 3*C*), and no apparent shrinkage of skeletal myotubes was observed after ouabain stimulation (Fig. 5*B*).

**Figure 6:**
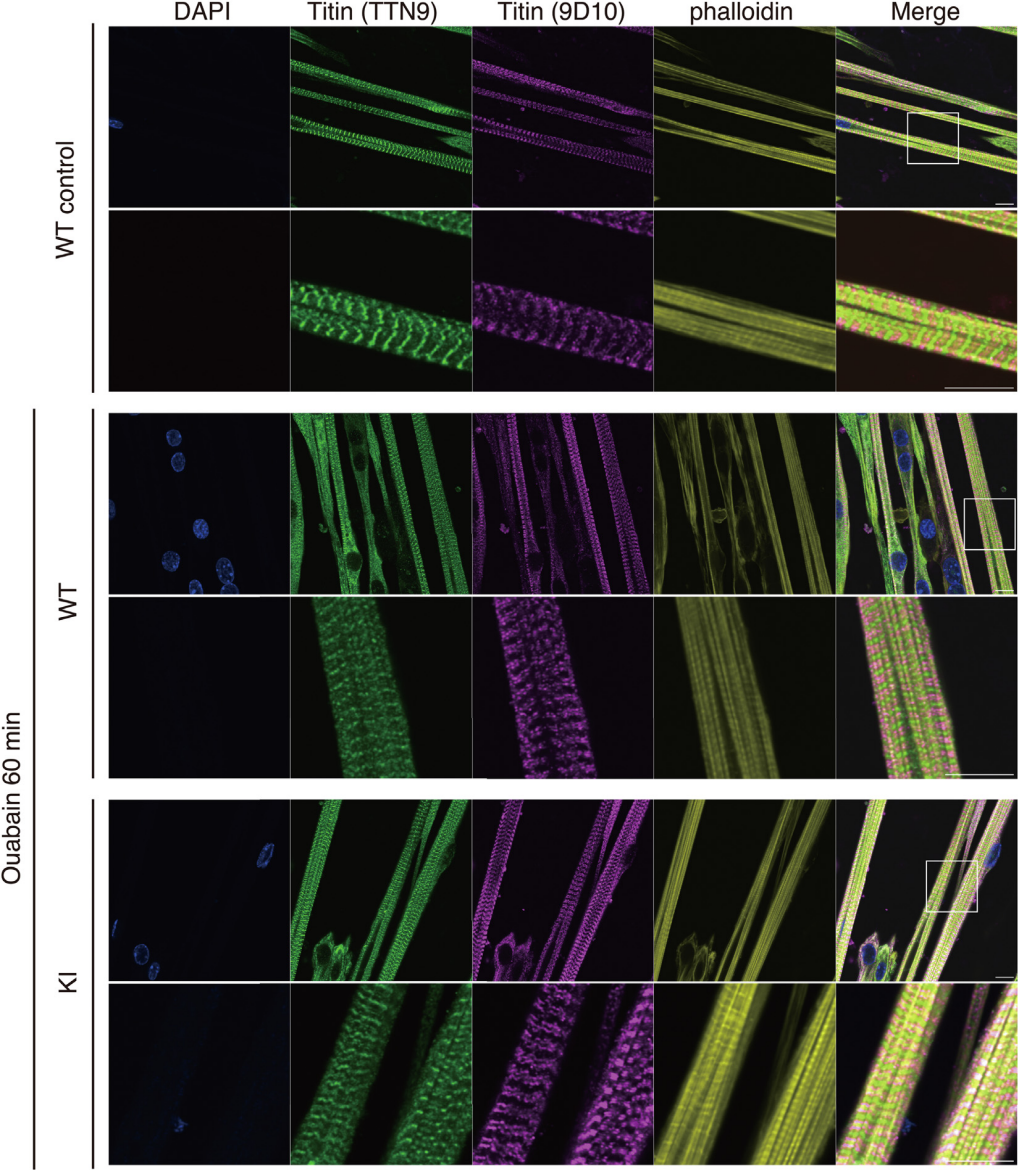
**Immunostaining of titin before and after ouabain stimulation**. Immunostaining of titin (TTN9, N2A, *green*) and titin (9D10, M-bands, *magenta*) in cultured skeletal muscles from WT and KI mice before and after stimulation with 1 mM ouabain for 60 min. Phalloidin: *yellow*. The *lower* panels show magnified images of the square area in the upper images. The scale bar represents 10 μm.

We further examined the subcellular localization of titin, a giant protein to which CAPN3 binds, in the sarcomere of skeletal muscles. Two antibodies, 9D10 and TTN9, against two distinct regions of titin around the N2A and M-bands, were used, respectively. In contrast to the drastic change in CAPN3 localization after ouabain stimulation, no apparent changes in the titin signal patterns were observed in the sarcomeres of skeletal muscles from either WT or KI mice after ouabain stimulation (Fig. 6). These data suggest that the translocation of CAPN3 from the M-band to the cytosol did not depend on titin distribution.

### Endogenous CAPN3 digests spectrin and talin in cultured skeletal muscles upon activation

**Figure 7:**
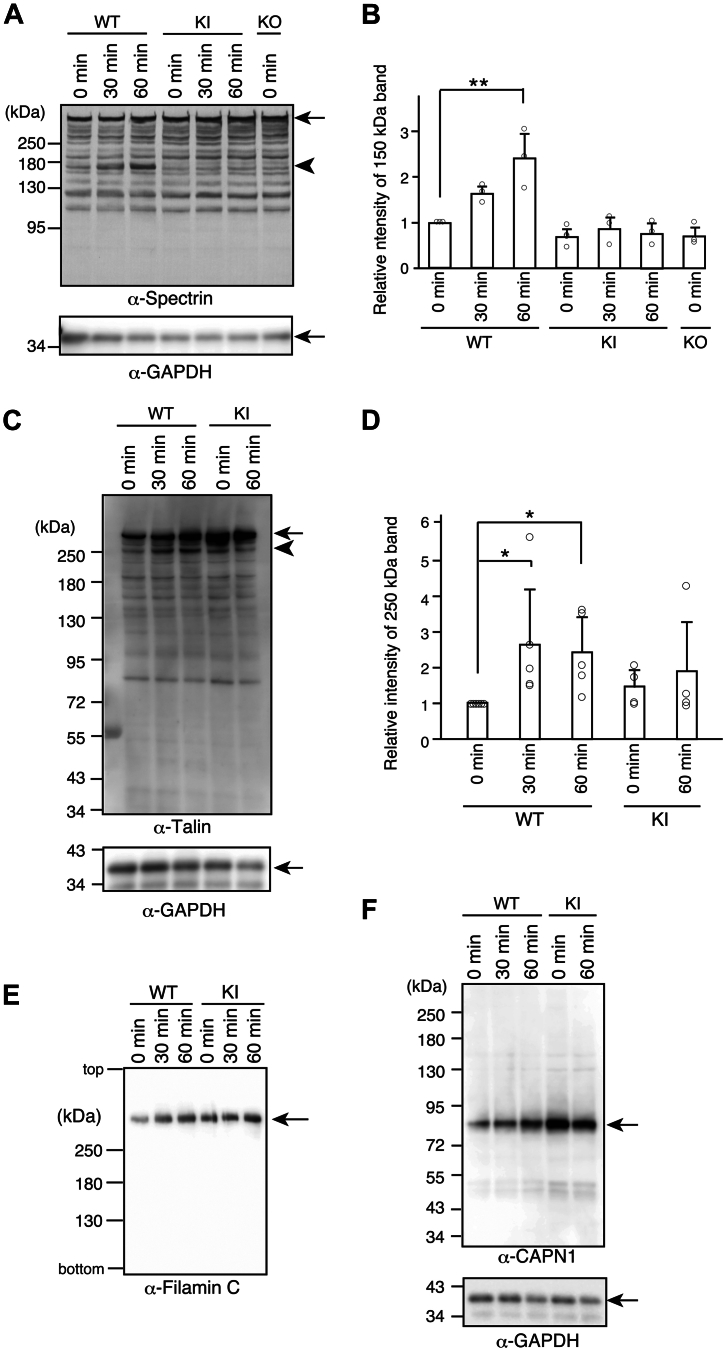
**Digestion of spectrin and talin by activated CAPN3 in cultured skeletal muscles upon ouabain stimulation**. *A*, time-dependent increase in the spectrin fragment (∼150 kDa) of cultured skeletal muscles from WT, but not KI and KO mice upon 1 mM ouabain stimulation. *Upper arrow* indicates the full-length of spectrin, whereas *lower arrowhead* indicates the cleaved 150 kDa fragment (*upper* panel). The same membrane was probed with antibodies for GAPDH (*lower panel*). *B*, relative band intensities of the 150 kDa spectrin fragments in (*A*). Mean ± SD. Number of experiments were 3 ∼ 4. ∗∗*p* < 0.01, one-way ANOVA with Dunnett’s multiple comparison test. *C*, time-dependent cleavage of talin in WT myotubes by ouabain. The same membrane was probed with antibodies for talin (*upper* panel) and GAPDH (*lower* panel). The *upper arrow* shows full length of talin and the *lower arrowhead* indicates the cleaved talin. *D*, relative intensity of the cleaved band (∼250 kDa) of talin. Mean ± SD. Number of experiments were 4 ∼ 6. ∗*p* < 0.05, *Steel-Dwass* test. *E*, expression of Filamin C in the cell lysates of ouabain-treated WT and KI myotubes. Samples were electrophoresed on a 6.0% SDS-PAGE and continued running for an additional 60 min after the dye front reached the bottom of the gels, as previously reported (59). Top and bottom indicated the top and bottom of the gel, respectively. *F*, immunoblot of CAPN1 in the cell lysates of ouabain-treated WT and KI myotubes. Autolysis of CAPN1 was not observed in ouabain-treated myotubes. The same membrane was probed with antibody against GAPDH (*lower* panel). The experiments were performed three times. CAPN3, calpain 3.

Several *in vitro* studies have identified potential substrates for CAPN3 (11, 18, 19, 20, 21, 22, 23, 24, 25). However, no reports have demonstrated endogenous CAPN3 digests substrates in intact skeletal myotubes in response to any stimuli. Therefore, we examined the processing of several potential substrates by CAPN3 in response to ouabain treatment. We examined the cytoskeletal proteins spectrin, talin, and filamin C, which are the most likely substrates identified so far (19, 26). A ∼150 kDa spectrin fragment in WT skeletal myotubes was detected after ouabain stimulation, but not in KI myotubes (Fig. 7*A*). Moreover, the relative intensity of the ∼150 kDa spectrin fragment increased in a time-dependent manner (Fig. 7*B*). Additionally, a time-dependent increase in ∼200 kDa talin fragments in WT skeletal myotubes was observed after ouabain stimulation, but this was not seen in KI myotubes (Fig. 7, *C* and *D*). In contrast to these two cytoskeletal proteins, the processing of filamin C in the lysates of ouabain-stimulated WT myotubes could not be detected using western blotting (Fig. 7*E*).

Considering both CAPN1 and CAPN3 are expressed in skeletal muscles and CAPN1 digests spectrin (27), we examined whether CAPN1 was activated in ouabain-treated cells. Although CAPN1 undergoes N-terminal autolysis for its activation, resulting in reduced molecular size from 80 to 76 kDa (28, 29, 30), no size reduction in CAPN1 occurred in ouabain-treated cells, suggesting that CAPN1 was not activated in the cultured skeletal muscles upon ouabain treatment (Fig. 7*F*). Taken together, these data strongly suggest that spectrin and talin are genuine substrates of endogenous CAPN3 in living skeletal myotubes.

## Discussion

In this study, we developed an antibody that specifically recognizes the autolysis of CAPN3, an essential event for CAPN3 activation. This antibody enabled the detection of the activation-dependent translocation of CAPN3 in cultured mouse skeletal myotubes; resting CAPN3 localized at the M-bands was activated and translocated into the cytoplasm after ouabain stimulation (Fig. 5). Notably, ouabain-induced CAPN3 activation depends on the long-lasting minimal increase in cytosolic Ca^2+^ levels, sufficient to trigger CAPN3 activation but not CAPN1. Cytosolically translocated CAPN3 digests at least two cytoskeletal proteins, spectrin and talin, as endogenous substrates in cultured skeletal myotubes (Fig. 7). No increase in the degradation of spectrin and talin was observed in KI myotubes, suggesting that spectrin and talin are the actual targets of endogenous CAPN3. Taken together, we succeeded in capturing the activation of CAPN3 and the subsequent digestion of spectrin and talin in cultured mouse skeletal myotubes upon ouabain stimulation. Although ouabain was not a relevant stimulus to trigger CAPN3 activation *in vivo*, our data improved our understanding of CAPN3’s subcellular dynamics and endogenous CAPN3 substrates in mouse skeletal muscles.

The presence of IS1 within the protease domain renders most CAPN3 inactive in skeletal muscle, and the precise mechanism of CAPN3 activation is unclear (10). The only physiological condition shown to induce *in vivo* CAPN3 autolysis is eccentric contraction (ECC), during which muscles are stretched while contracting. Murphy *et al*. reported that the activation of CAPN3, but not CAPN1, was observed following a single bout of eccentric exercise in human skeletal muscles (31), whereas all-out sprint exercise did not cause either CAPN3 or CAPN1 activation (32). Sequential CAPN3 and CAPN1 autolysis has been observed in skeletal muscles exposed to 200-repeated ECC in rats *in vivo* (33). Importantly, following ECC, a small but long-lasting increase in resting intracellular Ca^2+^ concentration (*e*.*g*., for hours and days) occurred in the isolated skeletal muscles of mice subjected to downhill treadmill running exercises (34) and in *in vivo* eccentric contractions (35). Given that CAPN3 autolysis is highly dependent on intracellular Ca^2+^ levels and occurs upon exposure to low Ca^2+^ concentrations (∼200 nM to 2 μM) for 60 min in skinned muscles (17, 36), fluctuations in cytosolic Ca^2+^ levels following ECC are likely associated with CAPN3 autolysis in skeletal muscles. In the present study, ouabain induced CAPN3 autolysis, which caused a gradual and slight increase in the resting Ca^2+^ concentration in both HeLa cells (Fig. 3*C*) and cultured skeletal myotubes (Fig. 5*D*). Preloading of intracellular Ca^2+^ chelator, BAPTA-AM inhibited ouabain-induced CAPN3 autolysis in HeLa cells, indicating a prerequisite of Ca^2+^ increase for ouabain-induced CAPN3 autolysis (Fig. 3*E*). Additionally, cytosolic Ca^2+^ elevation induced by ouabain did not reach a concentration that is sufficient for CAPN1 activation in cultured skeletal myotubes (Fig. 7*F*). CAPN1 activation requires approximately 5-fold higher Ca^2+^ concentration (5 μM) than CAPN3 activation (∼1 μM) (17); therefore, we reasoned that ouabain-induced cytosolic Ca^2+^ increase in cultured skeletal myotubes in our study was < 5 μM. Similar selective CAPN3 activation has also been reported in human skeletal muscles following eccentric exercise (31), although it is dependent on the strength of the exercise tasks (33, 37). Thus, although ouabain is not a physiological stimulus in skeletal muscles, it is an ideal pharmaceutical tool that imitates the small, long-lasting Ca^2+^ increase in skeletal muscles after ECC, required for CAPN3 autolysis in skeletal myotubes. Potential CAPN3 substrates were identified in cells exogenously expressing CAPN3 and the substrates or in cell lysates *in vitro* (21). Because ouabain treatment selectively induces the activation of endogenous CAPN3, identifying actual CAPN3 substrates in skeletal myotubes is important. The high degree of CAPN3 autolytic activation by ouabain can offer a more practical method to identify its target proteins, as opposed to the low percentage of CAPN3 autolysis observed following ECC (∼20%) (33, 38). These observations are strongly supported by our present finding of a distinct spectrin fragment in WT skeletal myotubes but not in KI myotubes (Fig. 7*A*), despite the fact that both CAPN1 and caspase are known to digest spectrin (39).

The mechanism by which ouabain increases the resting Ca^2+^ levels in HeLa cells and cultured skeletal muscle is unknown and beyond the scope of the present study. The most probable pathway is the activation of the Na^+^/Ca^2+^ exchanger, triggered by an increase in the cytosolic Na^+^ level due to Na^+^/K^+^ pump inhibition by ouabain. Notably, the binding of ouabain to the Na^+^/K^+^ pump can also trigger various signaling pathways, such as the phospholipase C/IP_3_ receptor/Ca^2+^ signaling pathway, activation of tyrosine kinases, and the Ras-Raf-ERK signaling pathway (40, 41, 42, 43). Considering that CAPN3 autolysis is dependent on the presence of ATP in addition to Ca^2+^ levels in skinned muscles (36), these signaling pathways may also contribute to the efficient CAPN3 autolysis by ouabain stimulation. We are currently investigating the ouabain-dependent modification of CAPN3 and its physiological role in CAPN3 autolysis in skeletal myotubes.

Previous studies, including ours, have reported several subcellular localizations of CAPN3 in the M-band, N2A, and Z-band of the sarcomeres, costameres, and myotendinous junctions using immunostaining with various CAPN3 antibodies (6, 11, 44, 45, 46, 47, 48). However, no studies have verified the specificity of the antibodies using CAPN3 KO and knockdown cells. According to Kramerova *et al*., all antibodies used for immunohistochemistry were capable of staining CAPN3 KO muscles (49). It is important to note that we performed thorough validation of available CAPN3 antibodies to disregard antibodies with such problems. We confirmed the specificity of the CAPN3 antibody (Proteintech, 28476-1-AP) used in this study for CAPN3 KO myotubes and demonstrated that CAPN3 was predominantly localized at the M-bands in the resting cultured myotubes (Figs. 4*C* and 5*A*). The residual cytosolic CAPN3 signals in the resting myotubes could be attributed to developmentally immature myotubes in our culture, as we also detected predominant CAPN3 signals at the M-bands in adult skeletal muscle tissues during the resting state (Fig. 4*E*). Although CAPN3 binding to the N2A region of titin is thought to be stronger than that of its binding to the M-bands *in vitro* (45), we did not observe clear CAPN3 signals around the N2A regions of resting WT skeletal myotubes. We could not completely rule out the possibility of subcellular localizations of CAPN3 at the N2A and Z-bands in resting skeletal muscles; nevertheless, their percentages were negligible, even if CAPN3 existed at those sites. Moreover, there exist a possibility that the antibody used in this study could not access the CAPN3 localized at the N2A and Z-bands. However, this is unlikely because exogenously expressed EGFP-tagged CAPN3 is also predominantly localized at the M-bands in resting cultured skeletal muscles (48). Thus, our data indicate that CAPN3 predominantly localizes to M-bands in resting skeletal muscles. Given that immunocytochemistry depends on sample conditions, for example, fixation and permeation methods, researchers should confirm the specificity of previously used antibodies for immunostaining using CAPN3-deficient samples.

Following ouabain stimulation, CAPN3 signals were predominantly observed in the cytoplasm of the cultured skeletal myotubes (Fig. 5*B*). Some AIS1 signals were observed in a striated pattern, suggesting that CAPN3 undergoes activation at the M-bands after ouabain stimulation. Moreover, protease-deficient CAPN3 was stable even after ouabain stimulation. Therefore, CAPN3 is translocated in an autolysis-dependent manner. During the initial 60 min of ouabain treatment, there was no apparent accumulation of CAPN3 signals in specific regions within the sarcomeres, including the N2A and Z-band regions. Thus, we concluded that CAPN3 is activated at the M-bands and translocates into the cytoplasm of skeletal muscles upon ouabain stimulation. Our findings are consistent with a recent study showing that CAPN3 dissociates from its tight binding to titin and diffuses upon activation, following the complete removal of IS1 (17).

Although the precise physiological function of CAPN3 in skeletal muscles remains unclear, increasing evidence suggests that CAPN3 has both proteolytic and nonproteolytic functions in skeletal muscles. CAPN3 KO mice exhibit progressive muscular dystrophy with abnormal sarcomere organization, misaligned thick filaments, and disorganized mitochondria (49, 50). In contrast, CAPN3:CS KI mice showed greatly improved sarcomere organization, but still display mild muscular dystrophy with central nuclei and myofiber splitting (48). The M-band has been proposed to play a crucial role in the stability of activated sarcomeres (51, 52). Given that CAPN3 predominantly localizes at the M-bands in its inactive form through interaction with titin, and as misaligned thick filaments were only observed in skeletal muscle from CAPN3 KO but not CAPN3:CS KI mice, we hypothesize that CAPN3 participates in the stabilization of the M-bands, although further studies will be necessary.

What is the role of the autolytic activity of CAPN3? We propose that one of the roles of CAPN3 autolytic activity is for its translocation. CAPN3 translocates from the M-bands to the cytoplasm when subjected to a small, sustained Ca^2+^ increase by ouabain, whereas the protease-dead CAPN3 mutant did not (Fig. 5*B*). Although the precise mechanism of CAPN3 translocation from myofibrils is unknown, the breakdown and dispersion of titin into the cytoplasm could not account for the mechanism of CAPN3 translocation into the cytoplasm, as immunosignals for titin using antibodies against two distinct regions around the N2A and the M-bands remained stable following ouabain stimulation (Fig. 6). Any deletion of CAPN3 results in the loss of its binding to the COOH terminal region of titin in the yeast two-hybrid system (53). Therefore, the removal of the IS1 region by autolysis could potentially decrease the affinity of the activated form of CAPN3 for titin and facilitate its translocation into the cytoplasm of skeletal myotubes after ouabain stimulation. Another importance of the autolytic activity of CAPN3 in the skeletal muscles is to activate CAPN3 for the modification of target proteins by partial digestion. Given that the activated form of CAPN3 translocates from the M-bands into the cytoplasm of sarcomeres, various proteins within the sarcomere compartments could be potential CAPN3 substrates. In this study, we confirmed that spectrin and talin, which function as linkages between the cytoskeleton and the sarcolemma membrane, are digested by endogenous CAPN3 in cultured skeletal muscles upon ouabain stimulation (Fig. 7, *A* and *C*). CAPN3 has much higher sensitivity to low cytosolic Ca^2+^ levels than CAPN1. Therefore, CAPN3 may be the most rational sensor for detecting small but long-lasting increases in resting Ca^2+^ levels, through which CAPN3 could initiate sarcomere remodeling *via* the partial digestion of several cytoskeletal proteins (19, 54, 55). It is also possible that CAPN3 translocation from the M-bands, itself, may affect the stability of the M-bands, which may contribute to the sarcomere remodeling. Further analysis of the different nature of WT and KI skeletal myotubes in the recovery phase following ouabain treatment would help understand the physiological signals triggered by partially digested CAPN3 substrates in living skeletal myotubes.

In summary, we developed a unique antibody capable of differentiating into the active form of CAPN3 from its inactive form in living skeletal myotubes. In combination with a commercially available and verified anti-CAPN3 antibody, we showed the decreased autolytic activity of some LGMDR1 mutants in exogenously expressed HeLa cells by immunostaining with our anti-AIS1 antibody. In addition to conventional western blotting, this method would be valuable for understanding the causal mechanism and diagnosing LGMDR1 mutations. In addition, we demonstrated autolysis-dependent subcellular translocation of CAPN3 from the M-bands into the cytoplasm of cultured skeletal myotubes, which required a small but long-lasting cytosolic Ca^2+^ increase by ouabain. Furthermore, activated CAPN3 digested the cytoskeletal proteins spectrin and talin in skeletal myotubes. Thus, our findings suggest that the protease activity of CAPN3 is necessary for its translocation from the M-bands to the cytoplasm as well as the subsequent digestion of at least two cytoskeletal proteins, spectrin and talin, in intact skeletal myotubes. Almost complete autolysis of CAPN3, but no obvious CAPN1 activation, in ouabain-treated cultured cells provides a suitable platform for the analysis of CAPN3-mediated signaling in living cells and will also open a new avenue for understanding the pathogenesis of LGMDR1.

## Experimental procedures

### Construction of expression vectors

The construction of pSRD-human CAPN3 and CAPN3:CS has been described previously (56). Mutations in pSRD-CAPN3 were introduced by site-directed mutagenesis using KOD Plus DNA polymerase (Toyobo) and appropriate primers in the Table S1. The sequences were confirmed by DNA sequencing.

### Antibodies

The antibodies used in this study include rabbit anti-CAPN3 polyclonal antibody (Proteintech, 28476-1-AP, antigen: 488–666 aa encoded by BC146672, 1:1000), anti-α-actinin antibody (Sigma-Aldrich, EA-53, 1: 1000), anti-spectrin alpha chain (nonerythroid) antibody (Sigma-Aldrich, MAB1622, clone AA6, 1:1000), anti-talin antibody (Sigma-Aldrich, T3287, 1:1000), anti-β-actin (MBL, M177–3, 1:1000), and filamin C (Myomedix Ltd, FLC-1, rabbit, 1:1000). A goat anti–CAPN3-IS2 polyclonal antibody (1:500) (57) was used for western blotting (in Fig. S2).

### Production of antibody against an AIS1

Two rabbits were immunized with a KLH-conjugated synthetic peptide (“CGTNMTY”) at a company (SCRUM Inc). The immunized rabbit sera were loaded onto an affinity column containing the SulfoLink Coupling Resin (Thermo Fisher Scientific K.K) cross-linked to a synthetic peptide “CGTNMTYGTS” to absorb autolysis-independent antibodies. The flow-through was further loaded onto the SulfoLink Coupling Resin linked to “CGTNMTY” peptide. After washed with 25 mM Tris–HCl (pH = 7.5) containing 0.5 M NaCl, the anti-AIS1 antibody against the COOH terminus of the “CGTNMTY” peptide was eluted with 0.1 M glycine-HCl (pH = 2.5) and was quickly neutralized with 1M Tris–HCl (pH = 8.5).

### Labeling of anti-CAPN3 and anti-AIS1 antibodies with fluorescent dye

Rabbit anti-AIS1 and rabbit anti-CAPN3 antibodies (Proteintech, 28476-1-AP) were cross-linked to a fluorescent dye (CF Dye) using Mix-n-Stain CF488A Antibody Labeling Kit (Biotium, Inc, 92253) and Mix-n-Stain CF555 Antibody Labeling Kit (Biotium, Inc, 92254), respectively, according to the manufacturer’s instructions.

### Myoblast culture

The experimental animals were handled according to the guidelines of the Experimental Animal Care and Use Committee of the Tokyo Metropolitan Institute of Medical Science (approval number: 24–034). The *Capn3* KO and *Capn3* KI mice (48) were maintained at a specific pathogen free facility that kept rooms at 23 ± 1 °C and 50 ± 10% humidity, under a standard 12-h light/12-h dark cycle. Myoblasts were isolated as previously described (58) with slight modifications. Briefly, skeletal muscles (femurs) from 4 to 5-week-old male mice were minced into small pieces using scissors. The pieces were incubated at 37 °C with 1 ml of enzymatic solution (PBS containing 500 U/ml collagenase type II; Sigma-Aldrich), 1.5 U/ml collagenase D (Sigma-Aldrich, 11088858001) 2.5 U/ml dispase II (Gibco/Thermo Fisher Scientific, 17105–041), and 2.5 mM CaCl_2_ in 50 ml tubes for 60 min in a water bath, while agitating the tube every 10 min. Following the addition of 30 ml PBS, the samples were centrifuged at 300g for 5 min, and the pellets were resuspended in 2 ml of proliferation medium (PM) (Dulbecco's modified Eagle medium [DMEM] containing 20% fetal bovine serum [JRH Biosciences/Sigma-Aldrich, 12603C], 10% horse serum [Biowest, S0900], 1% chicken embryo extract [US Biological, C3999], 5 ng/ml bFGF [Fuji film, 064–05381], and penicillin–streptomycin [Nacalai tesque, 09367–34]). The suspensions were seeded in one well of a six-well dish, precoated with 10 times-diluted Matrigel (Corning356234). After 3 days of incubation at 37 °C and 5% CO_2_, the well was washed twice with 2 ml of PBS, and the cells were detached with 0.25% trypsin/EDTA, filtrated with pluriStrainer 15 μm (pluriSelect, 43–50015–03), and maintained in PM medium in 10 cm collagen I coat dish (AGC Techno Glass Co, Ltd, 4020–010).

For differentiation, the cells (at a density of ∼2.0 x 10^5^) in PM were plated in 3.5 cm dishes coated with 20 times diluted Matrigel overnight. The medium was subsequently changed to differentiation medium (DMEM containing 5% horse serum and penicillin–streptomycin), next day. To examine ouabain-induced CAPN3 autolysis, cells at day 7 after differentiation were treated with a differentiation medium containing 1 mM ouabain (Sigma-Aldrich, O3125) for the indicated time periods and lysed with lysis buffer. The lysates were centrifuged at 20,630g for 10 min at 4 °C, and the protein concentration of the supernatants was measured using a DC protein assay (Bio-Rad, 5000111). Approximately, 20 μg of protein was loaded into each well of an SDS-gel for western blotting analysis. For immunostaining and Ca^2+^ imaging, ∼5.0 x 10^4^ cells were plated and differentiated for 7 days on 0.1% polyethylenimine/Matrigel (1/20)-coated 12 mm coverslips (Paul Marienfeld GmbH & Co. KG, Deckglaser cover glass 0111520) in 24-well dishes.

### Transfection and immunoblotting

HeLa, COS-7, and HEK 293T cells were cultured in DMEM (Nacalai Tesque, 08458–16) supplemented with 10% fetal bovine serum, Penicillin-Streptomycin Mixed Solution [stabilized] (Nacalai Tesque, used at a working concentration of 100 units/ml of penicillin, and 100 μg/ml of streptomycin). The cells (∼5 x 10^5^) were cultured in a 6 cm dish and were transfected with appropriate plasmids (1 μg) using 3 μg of PEI-Max 40k (Polysciences Inc,) on the next day. After 16 ∼20 h of the transfection, the cells were lysed using 700 μl lysis buffer (10 mM Tris–HCl pH 7.0, 150 mM KCl, and 2 mM EGTA-KOH pH 8.0) containing cOmplete Protease Inhibitor Cocktail (Sigma-Aldrich) at 4 °C for 15 min. The lysates were centrifuged at 20,630g for 10 min, and the supernatants were mixed with 4x SDS-PAGE sample buffer and boiled at 95 °C for 5 min. To examine the effect of intracellular Ca^2+^ chelation on ouabain-induced CAPN3 autolysis, the cells were incubated with the medium containing 25 μM BAPTA-AM (Dojindo) at least 5 min before ouabain stimulation, and ouabain stimulation was performed in the presence of BAPTA-AM.

The proteins were separated using Extra PAGE One Precast Gel 7.5 to 15% (Nacalai tesque, 13065–54) and Extra PAGE One Precast Gel 5 to 10% (Nacalai Tesque, 13059–44) and transferred to a polyvinylidene difluoride transfer membrane. To detect filamin C, the samples were electrophoresed on a 6.0% SDS-PAGE and continued running for an additional 60 min after the dye front reached the bottom of the gel, as previously reported (59). The membrane was blocked with PBS/0.05% Tween 20 (PBST) containing 3% skim milk for 60 min and was then treated with appropriate antibodies for 1 h at room temperature (RT). After washing with PBST for 15 min, the membranes were probed with horseradish peroxidase-conjugated secondary antibodies for 1 h. Next, the membrane was washed with PBST for 15 min, and protein bands were visualized using Immobilon Western Chemiluminescent HRP Substrate (Millipore) on a LAS3000 (Fujifilm). The images were analyzed using Fiji open-source software (60), and the band intensities were normalized to those of WT samples at 0 min.

### Immunocytochemistry

For immunostaining of cultured cells, the cells were washed with PBS and fixed with 4.0% PFA/PBS for 10 min at RT. After washing with PBS, the cells were permeabilized with 0.2% Triton/PBS for 5 min at RT and blocked with 5% goat serum/PBS for 30 min at RT. The cells were probed with CF488-linked anti-AIS1 and CF555-linked anti-CAPN3 (Proteintech, 28476-1-AP) antibodies diluted in Can Get Signal immunostain Solution A (Toyobo) for 1 h at RT (for HeLa and COS-7 cells) or at 4 °C overnight (for cultured myotubes). After washing with PBS for 30 min, the cells were mounted with Vibrance Antifade Mounting Medium with 4′,6-diamidino-2-phenylindole (Vector Laboratories, IncH-1800) and observed under a confocal fluorescence microscope (FV3000) (Evident Corporation). The images were analyzed using Fiji open-source software. The fluorescence intensities of individual HeLa cells were measured with the region of interest positioned over the cytosolic region of the cells.

### Immunohistochemistry

The mice (7–8 months old) were sacrificed by cervical dislocation, and the skeletal muscles were carefully dissected using forceps. The tissues were immersed in 4.0% PFA/PBS for 3 h at 4 °C. Subsequently, the tissues were immersed in 30% sucrose/PBS at 4 °C overnight, and subsequently embedded in O.C.T. compound (Sakura Finetek Japan Co, Ltd) using prechilled isopentane cooled with liquid nitrogen. The frozen sections were then cut into 10 microns thick sections using cryostat CM1950 (Leica) and attached on glass slide.

The sections were immersed in PBS, permeabilized with 0.2% Triton/PBS for 5 min, and subjected to antigen retrieval using boiled 10 mM Tris–HCl (pH 9.0) for 10 min. After washing with PBS, the sections were blocked with PBS containing 5.0% bovine serum albumin for 1 h at RT. Then, the sections were probed with appropriate antibodies (anti-CAPN3 antibody (Proteintech, 28476-1-AP, 1:500); anti-actinin (Sigma-Aldrich, EA-53, 1:500)) diluted in the appropriate blocking buffer at 4 °C overnight. After being washed three times with PBS for a total of 30 min, the sections were probed with Alexa Fluor 488 goat anti-rabbit IgG (H + L) cross-adsorbed secondary antibody and Alexa Fluor 594 conjugated anti-mouse IgG antibody (Thermo Fisher Scientific, A-11034 and A11032, 1:1000) for 30 min at RT. After washing with PBS for 30 min, the sections were mounted with Vibrance Antifade Mounting Medium with 4′,6-diamidino-2-phenylindole (Vector Laboratories, Inc, H-1800) and observed under a confocal fluorescence microscope (FV3000) (Evident Corporation).

### Ca^2+^ imaging

Ca^2+^ imaging was performed as described previously with modifications (61). HeLa cells (∼2 x 10^4^/cm^2^) and skeletal myotubes (day 7 after differentiation) cultured on 12 mm coverslips were loaded with 5 μM Fura 2 AM (Dojindo) in recording solution (115 mM NaCl, 5.4 mM KCl, 1 mM MgCl_2_, 2 mM CaCl_2_, 20 mM Hepes, and 10 mM glucose, pH 7.42) for 30 min at RT. After washed with recording solution, Fura 2 signals were observed using an inverted microscope (IX80; Evident Corporation) equipped with a filter exchanging device (IX2-SHA, Evident Corporation, 340 ± 10 and 380 ± 10 nm excitation filters), a 400 nm dichroic beam splitter, and a band-pass emission filter at 510 to 550 nm. Fura 2 fluorescent images were captured every 30 s for 1 mM ouabain-treated cells and every 5 s for 10 μM ATP (Selleck Biotechnology, S1985)-stimulated cells with an EM-CCD digital camera (C9100–13; Hamamatsu Photonics K.K.) and were analyzed using an imaging software MetaMorph (Molecular Devices Japan K.K).

### *In vitro* protease assay of CAPN3

COS-7 cells transiently transfected with pSRD-CAPN3 or mutants were lysed in lysis buffer at 4 °C for 15 min and centrifuged at 20,630g for 10 min at 4 °C. The cell lysates were incubated with or without 5 mM Ca^2+^ for 2 h at 37 °C. Next, 4x SDS-PAGE sample buffer was added to the sample, and boiled for 5 min at 95 °C. The autolytic activity shown in Figure S2*B* was calculated as the ratio of the band intensity of full-length CAPN3 to that of the sum of the bands lower than 60 kDa.

### Statistical analysis

Statistical analysis was performed using EZR software (62). All data were expressed as mean ± SD, and individual data were indicated as scatter plots in the figures.

## Data availability

All data are contained within the manuscript.

## Supporting information

This article contains supporting information.

## Conflict of interest

The authors declare that they have no conflicts of interest with the contents of this article.
