# Supplementary material for: In situ detection of activation of CAPN3, a responsible gene product for LGMDR1, in mouse skeletal myotubes
Source: J Biol Chem. 2025 Apr 23;301(6):108536. doi: 10.1016/j.jbc.2025.108536 (PMC12148437; doi:10.1016/j.jbc.2025.108536)
Supplement: Supporting Information [file mmc1.pdf]

Supporting Information

Title: In situ detection of activation of CAPN3, a responsible gene product for LGMDR1, in mouse skeletal myotubes

Authors

Chihiro Hisatsune*, Fumiko Shinkai-Ouchi, Shoji Hata, and Yasuko Ono*

Calpain project, Tokyo Metropolitan Institute of Medical Science, 2-1-6 Kamikitazawa, Setagaya-ku, Tokyo, 156-8506, Japan

Contents

Figure S1. Detection of CF^{TM} dye-labelled anti-AIS1 and CAPN3 antibodies

Figure S2. Decreased autolysis of the LGMDR1 mutants within IS2

Table S1. Primers used in this study

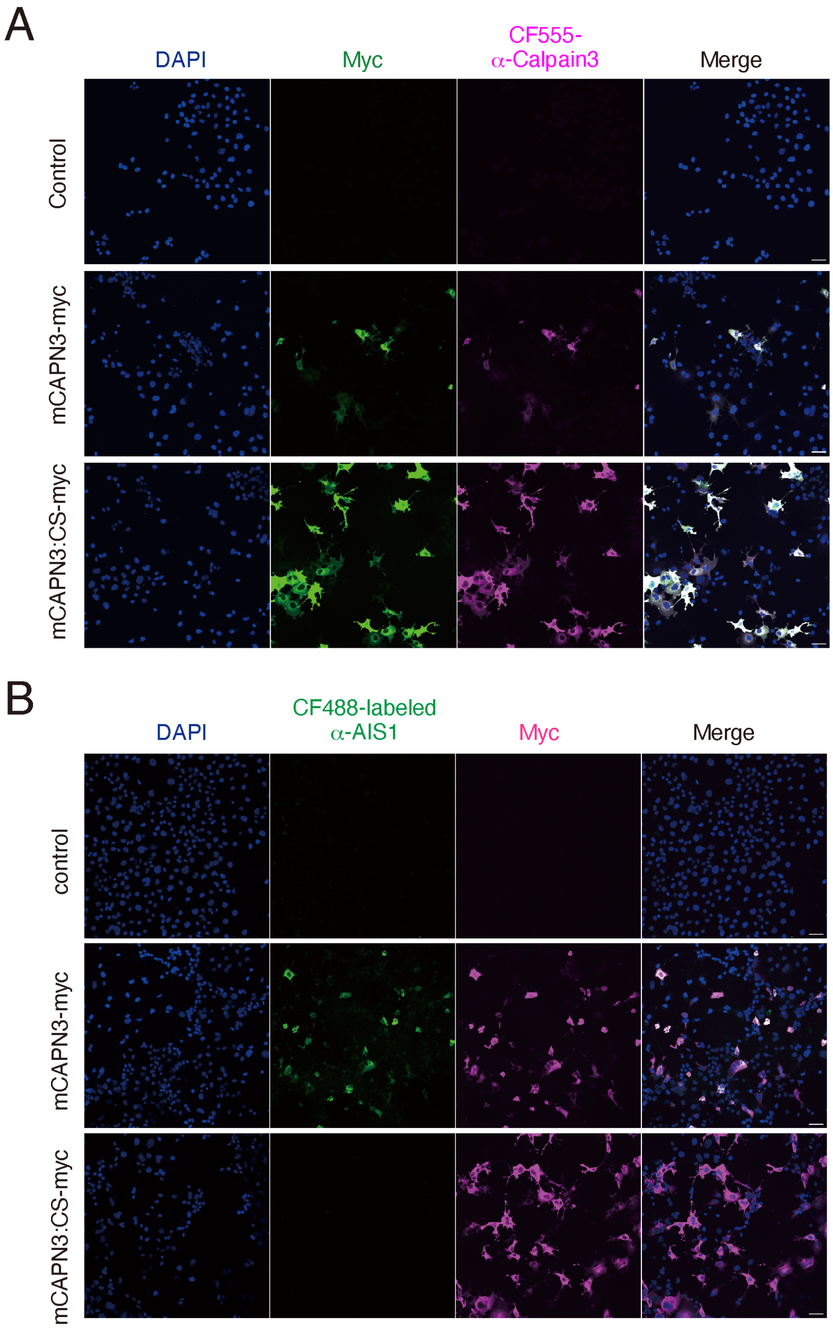

Figure S1. Detection of CF^{TM} dye-labelled anti-AIS1 and CAPN3 antibodies

(A) Immunostaining of Myc-tagged CAPN3 and CAPN3:CS expressed in HeLa cells using anti-Myc (green) and CF^{TM}555 Dye-conjugated anti-CAPN3 (magenta) antibodies. Scale bar: 50 µm.
(B) Immunostaining of Myc-tagged CAPN3 and CAPN3:CS expressed in HeLa cells using anti-Myc (magenta) and CF^{TM}488A Dye-conjugated anti-AIS1 (green) antibodies. Scale bar: 50 µm.

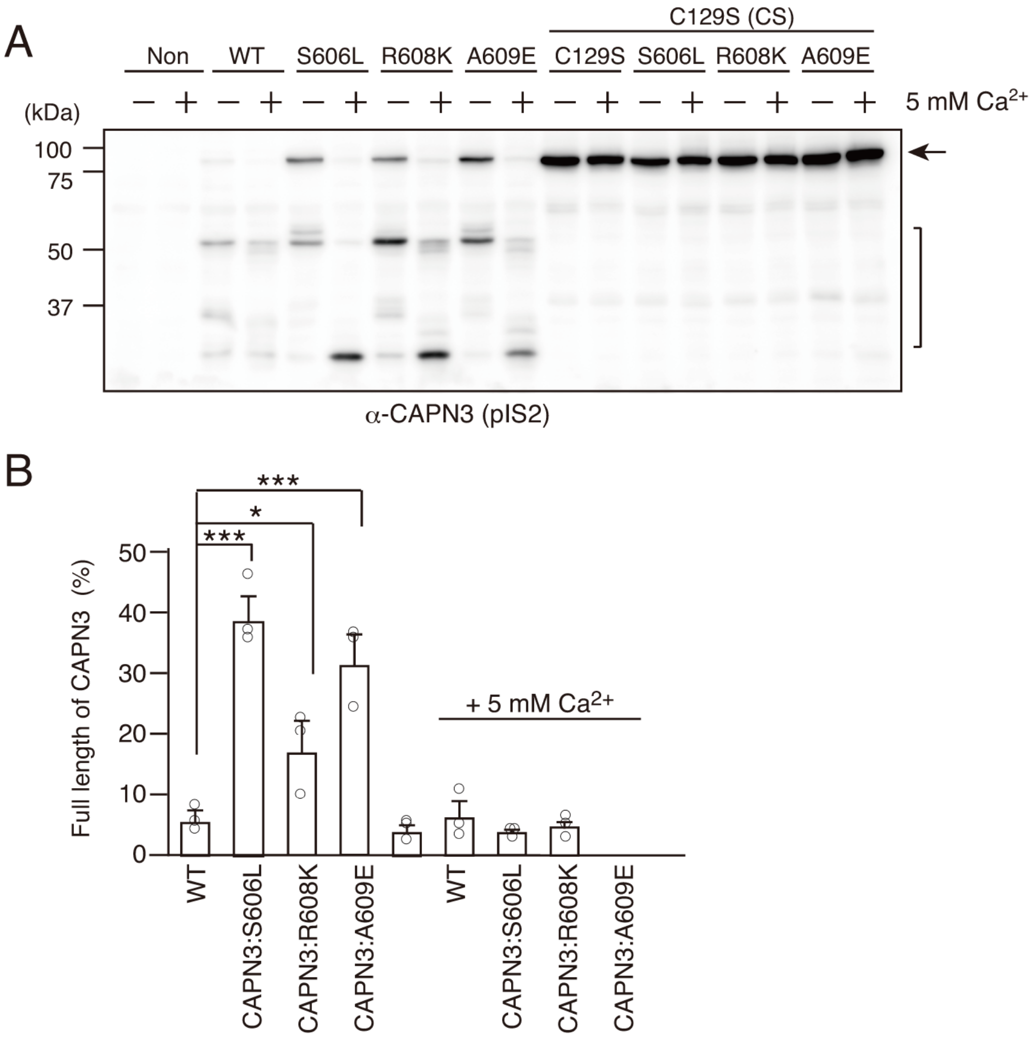

Figure S2. Decreased autolysis of the LGMDR1 mutants within IS2

(A) Autolytic activity of wild-type CAPN3 (WT) and its LGMDR1 mutants. COS-7 cell lysates transiently expressing CAPN3 and its LGMDR1 mutants were incubated at 37 ºC with or without 5 mM Ca^{2+} for 2 h. The cell lysates were probed with an anti-CAPN3 (pIS2) antibody. Compared to the WT, the mutants showed a clear band at 94 kDa in the absence of Ca^{2+}, suggesting that these LGMDR1 mutants (S606L, R608K, and A609E) exhibited weaker autolytic activity compared to the wild-type CAPN3. CAPN3:CS and its mutants do not undergo autolysis in COS-7 cells. The arrow indicates the position of full-length CAPN3. The parenthesis indicates the position of autolyzed fragments of CAPN3. (B) Percentage band intensity of full-length CAPN3 relative to the total band intensities including the fragments lower than 60 kDa in the panel A. Mean +/- SD. N= 3. ***$P < 0.001$, *$P = 0.0268$, *one one-way ANOVA with Dunnett's multiple comparisons test*.

Primer	Sequence
hCAPN3-S606L-S	catcttcgttttggacagagcaaac
hCAPN3-S606L-AS	gtttgctctgtccaaaacgaagatg
hCAPN3-R608K-S	cttcgtttcggacaaagcaaacagc
hCAPN3-R608K-AS	gctgtttgctctttccgaaacgaag
hCAPN3-A609E-S	gtttcggacagagaaaacagcaacaag
hCAPN3-A609E-AS	cttgttgctgttttctctgtccgaaac
hCAPN3-delta ex16-S	ccacagcctggcagctctgatcaggaaag
hCAPN3-delta ex16-AS	cttggtttttttctttttcactggccgatc

Table S1. Primers used in this study
